# Potential factors influencing COVID-19 vaccine acceptance and hesitancy among university students in Banglad*e*sh: a cross-sectional comparative study

**DOI:** 10.1017/S0950268822001820

**Published:** 2022-12-20

**Authors:** Debendra Nath Roy, Md. Shah Azam, Mohitosh Biswas, Ekramul Islam

**Affiliations:** 1 Department of Pharmacy, Jashore University of Science and Technology, Jashore-7408, Bangladesh; 2 Institute of Education and Research, University of Rajshahi, Rajshahi-6205, Bangladesh; 3 Department of Marketing, University of Rajshahi, Rajshahi-6205, Bangladesh; 4 Rabindra University, Shahjadpur, Bangladesh; 5 Department of Pharmacy, University of Rajshahi, Rajshahi-6205, Bangladesh

**Keywords:** Bangladesh, COVID-19, students, vaccine acceptance, vaccine hesitancy

## Abstract

This study investigated Coronavirus disease 2019 (COVID-19) vaccine acceptance, and compared the potential factors influencing vaccine acceptance and hesitancy between public university (PuU) and private university (PrU) students in Bangladesh. An anonymous, self-administered questionnaire was sent to 640 PuU and 660 PrU students in Google Form between 25th September and 22nd November 2021, which resulted in the participation of 1034 (461 PuU *vs.* 573 PrU) respondents (response rate: 72.03% *vs.* 86.81%). The pooled vaccine acceptance rates among PuU and PrU students were almost similar (88.1%, 95% confidence interval (CI) 85.1–91.1 *vs.* 87.6%, 95% CI 84.6–90.6). Employing binary logistic regression to assess the association between various potential factors and vaccine acceptance, the study revealed that out of 10 predictors, ‘safety’ and ‘efficacy’ had highly significant positive associations with vaccine acceptance in both cohorts (*P = 0.000, P = 0.005)*. ‘Political roles’ was found to have varied effects– a significant (*P = 0.02)* negative and a significant positive (*P* = 0.002) association with vaccine acceptance in PuU and PrU students, respectively. Additionally, ‘communication’ (*P = 0.003)* and ‘trust’ *(P = 0.01)* were found to have significant positive associations in PrU students while ‘rumours’ (*P = 0.03)* had negative association in PuU students. The odds of accepting the COVID-19 vaccine were 1.5 *vs.* 0.9 in PuU and PrU students. Although chi-square analysis did not show any significant association between gender and vaccine acceptance, discrepancies were found in the factors that potentially affect vaccine uptake decision between PuU and PrU students. COVID-19 vaccine uptake may be improved if vaccine-related information becomes available and is communicated to large numbers of people effectively. The implementation of multidisciplinary interventional educational programmes may also be considered as a preferred approach to improve student's engagement in pandemic awareness and vaccine readiness.

## Introduction

The Coronavirus disease 2019 (COVID-19) pandemic is not over yet. The death toll associated with COVID-19 is still considered a global health challenge, because all countries are encountering a unique public health crisis due to the rapid spread of infection around the world [1]. Although few repurposed drugs have shown clinical potential to reduce morbidity among COVID-19 infected individuals, no specific antiviral drugs have been approved [2]. The severity and pervasiveness of COVID-19 provoked the emergency use of an effective vaccine to control and gradually stop the pandemic. In the last few decades, vaccines have been among the most significant therapeutic interventions used in preventing the emergence and re-emergence of numerous infectious diseases [3]. Accordingly, the Centers for Disease Control and Prevention (CDC) declared vaccination as one of the top ten public health achievements [4]. Despite the proven benefits of immunisation, there still seems to be some significant doubt in the public regarding their willingness to accept COVID-19 vaccines. Vaccine hesitancy as well as missed opportunities remains recognised public health concerns, arising in relation to influenza vaccination [5], human papillomavirus (HPV) vaccination [6] and now for COVID-19 vaccination [7]. Reportedly, hesitancy towards or refusal of a vaccine refers to the unwillingness to take it, even when the service is available to deliver it [8]. The World Health Organization has denoted vaccine hesitancy as one of the top ten threats [9], and now COVID-19 vaccine hesitancy is a growing phenomenon among the various population sub-groups, and is showing substantial regional variability [10].

In Bangladesh, the pilot COVID-19 vaccination programme was inaugurated on 27th January, 2021, during which the government launched the biggest-ever mass vaccination programme aiming to vaccinate 80% of the country's total population [11]. Since vaccination has begun to be used, several studies focusing on COVID-19 vaccination among the adult and general population have reported relatively high vaccine hesitancy rates between 40% and 55% [12–16]. However, some other studies conducted in the same period have documented lower vaccine hesitancy rates, from 20% to 35% [17–24]. The prevalence of vaccine hesitancy is high among older people, the less educated, day labourers and chronically diseased individuals [24], and vaccine acceptance willingness was high in students [17] and young adults [18]. A cross-sectional study conducted in the middle of 2021 reported a 15.7% COVID-19 vaccine hesitancy rate among the rural population. The study concluded that vaccination safety, effectiveness data, and trust were the facilitators, while rumours were the barrier to implementing mass vaccinations in Bangladesh [25]. Focusing on the university education sector in Bangladesh, Hossain *et al*. (2021) [26] suggested a 27.3% COVID-19 vaccine hesitancy rate in public university (PuU) students. The author deduced female gender, low family income, non-infected individuals, poor knowledge, and negative perceptions of COVID-19 vaccines were characteristics that increased vaccine resistance [26]. In another study, Hoque *et al*. (2021) [27] recommended being more proactive in directing COVID-19 vaccination data and perceived health benefits towards the university students, because the study reported 27.7% hesitancy and 15.7% refusal intention among university students due to vaccine safety and effectiveness concerns [27].

Until recently, few studies have concentrated on COVID-19 vaccine acceptability among university students [26, 27] in Bangladesh, and a comparative analysis on COVID-19 vaccination consequences between public and private university students has yet to be performed. This study thus aimed to investigate COVID-19 vaccine acceptance intention, and to compare the potential factors influencing vaccine acceptance and hesitancy between public and private university students in Bangladesh.

## Materials and methods

### Study design

This cross-sectional comparative study applied a self-administered anonymous multi-item questionnaire. The questionnaire was deployed online using online survey tool (Google forms) and was conveniently sent to the students of different public and private universities between 25th September, 2021 and 22nd November, 2021, either via social media networks or personal emails. According to the latest census, in total, 51 public universities and 108 private universities were approved by the University Grants Commission of Bangladesh. The permission to conduct this study was obtained from the ‘Ethical Review Committee’ (IRC), Faculty of Biological Science and Technology, Jashore University of Science and Technology in Bangladesh. The detail research protocol was reviewed by the IRC before the study began. Data were collected and analysed anonymously; no clinical intervention was applied to the subjects. The Ethical Review Committee thus approved the study as exempt.

### Setting and participants

Government sponsored (public) and non-government sponsored (private) university students in Bangladesh. No financial or in-kind reward was offered to students who completed the survey.

### Participants' inclusion criteria

The eligibility criteria for the participants were the following: (i) to understand and agree to the study objectives and provide anonymous data on COVID-19 vaccine and vaccination; (ii) public or private university students in Bangladesh; (iii) studying in a bachelor's degree programme; and (iv) studying in a master's degree programme, and/or studying in a research degree programme. This study did not harm the individuals because no intervention was applied to the subjects. The individual was free to refuse participation.

### Measures and survey instrument development

The theoretical concept of global COVID-19 vaccine acceptance and hesitancy was conceptualised by Roy *et al*. (2022) [10]. The items of the validated questionnaire were adopted from a theoretical analysis of recent studies on COVID-19 vaccination conducted among diverse student groups worldwide. Alongside this, in-field consultation was carried out when designing the key items of the questionnaire. The questionnaire focused on multifaceted aspects of COVID-19 vaccination and its consequence, and was constructed in the English language. Each item in the preliminary questionnaire was content- and face- validated by a panel of several experts from reputed universities in Bangladesh, which ensures the relevance and clarity of the questionnaire. The revised questionnaire was subsequently pre-tested on 20 students, who were, later excluded from the final analysis.

The survey instrument assessed (1) the socio-demographic characteristics of the respondents; (2) the intention to uptake COVID-19 vaccines; and (3) factors influencing COVID-19 vaccine acceptance and hesitancy. A non-parametric data analytical tool (binary logistic regression) was employed to analyse the associations between predictor variables and the outcome variable in a 95% confidence interval (CI).

### Survey administration

The convenience sampling technique was used for gathering systematic data from online survey tools. This process created a survey with the goal of collecting maximum insights from the sample of entities for the purpose of developing quantitative variables of the attributes. To avoid potential sources of non-response bias, the online questionnaire was distributed among almost all the students of the universities, and encouraged them to participating in this study.

### Study variables

As the response variable of the study, we measured willingness to uptake a vaccine and the responses were measured as a binary variable (1 = Yes, 0 = No). The socio-demographic characteristics of the respondents were also noted. In analysing the data in a binary regression model, we investigated the impacts of several socio-psychological and vaccine-related factors on the outcome response variable dichotomised into 1 = Yes and 0 = No.

### Sample size calculation

Binary logistic regression was used, and for observational studies with large sample size, taking minimum sample sizes of 450–500 is necessary to derive the binary logistic regression statistics that represent the parameters. The other recommended rules of thumb are an event per variable of 50, and the formula: *n* = 100 + 50*i,* where *i* indicate to number of independent variables incorporated into the final model [28]. Pilot tests (*n* = 20) were conducted to assess the clarity of the survey items and to evaluate the average time of survey accomplishment.

### Equations for binominal regression

The general form of logistic regression is as follows:1
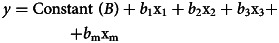
where y is the linear combination function. The computational algorithms are as follows:2
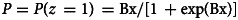


here, *P* is referred as the probability of vaccine uptake intention, x = vector of explanatory variables. Function of y is represented as logit (*P*), i,e., the log (to base e) of the odds or likelihood ratio that the dependent variable *z* is 1.3
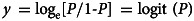


Usually equation (2) and (3) are written as logit (*P*) or the log odd ratio as follows-4
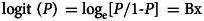


Individual coefficient (B) reflects the degree of influence of predictor variables to the outcome variable.

### Data analysis

Descriptive statistics expressed as weighted frequencies and percentages were applied on the categorical variables and socio-demographic characteristics. Binary logistic regression analysed the association pattern between predictor variables and outcome variables. The model was evaluated via the Nagelkerke *R*^2^ value. The goodness-of-fit was assessed using omnibus tests of model coefficients and Hosmer and Lemeshow tests [29]. Microsoft excel (version 10) was used for extracting the sample from Google Forms, and we then imported the data into SPSS. The entire analysis was conducted using the IBM SPSS statistical package (version 25). The minimum significance level (*P* vale) was set to 0.05. The online survey precludes the acceptance of any incomplete survey instrument, which ensures the collection of complete responses. Thus, no missing data were received.

## Results

### Respondents' characteristics

Table 1 displays the comparison of socio-demographic characteristics among the studied sets in the population. We checked for eligibility criteria and confirmed the eligible participants. A total of 461 PuU and 573 PrU students, who were potentially eligible, participated in and completed the survey. Most of the students were 20–24 years in age (81.6% *vs.* 91.6%) and were in the final year of their bachelor degree programme in final year bachelor degree programme (38.4% *vs.* 40%). Science students were the highest group in the PuU (40.6%), while business students were most prevalent in the PuU in PuU (39.8%). In total, 51.6% male participants were recruited from the PuU, whereas 52.2% females were recruited from the PrU. Most of the participants were Muslim by religion (88.3% *vs.* 87.6%).

**Table 1.** Comparative socio-demographic characteristics of the participants (*N* = 461vs.573)

Variables	Public university		Private university	
	*N*	*%*	*N*	%
Age distribution				
15–19 Years	15	3.3	10	1.8
20–24 Years	376	81.5	525	91.6
25–29 Years	66	14.3	34	5.9
30 Years & above	4	0.9	4	07
Year of study				
1st Year	31	6.7	29	5.1
2nd Year	61	13.2	85	14.8
3rd Year	110	23.8	180	31.4
4th Year	177	38.4	229	40.0
Masters and Research	82	17.9	50	8.7
Study branch				
Science	187	40.6	202	35.2
Arts & Humanities	126	27.3	143	25.0
Business	148	32.1	228	39.8
Home location (division)				
Dhaka	131	28.4	241	42.1
Rajshahi	123	26.7	139	24.3
Khulna	98	21.3	54	9.4
Chattogram	27	5.8	57	9.9
Mymensingh	23	5.0	19	3.3
Rangpur	44	9.5	42	7.3
Sylhet	9	2.0	9	1.6
Barishal	6	1.3	12	2.1
University location				
Metropolitan city	188	40.8	394	77.5
District city	221	47.9	95	16.6
Other	52	11.3	34	5.9
Gender				
Male	238	51.6	274	47.8
Female	223	48.4	299	52.2
Religion				
Muslim	407	88.3	502	87.6
Hindu	54	11.7	71	12.4
Experience of COVID-19				
Corona Infection positive	46	10.0	90	15.7
Vaccine intention				
Reservation to uptake vaccine	55	11.9	71	12.4
Accept vaccination anytime	406	88.1	502	87.6

### Results of descriptive statistics

Table 2 shows the descriptive statistics of the predictor variables and outcome variable in this study. The pooled COVID-19 vaccine acceptance rate was 88.1% (95% CI 85.1–91.1) in the PuU students and 87.6% (95% CI 84.6–90.6) in the PrU. Since the online survey precludes the acceptance of incomplete survey, the study's variable of interest produced no missing data.

**Table 2.** Descriptive statistics of the study's variable of interest

Variables	Public university		Private university	
Operational definition	Mean	s.d.	Mean	s.d.
I intend to accept vaccination anytime (Yes = 1, otherwise = 0)	0.88	0.325	0.88	0.330
I confirm that the vaccination is safe (Yes = 1, otherwise = 0)	0.79	0.405	0.81	0.393
I think that the vaccination has no significant side effect (Yes = 1, otherwise = 0)	0.54	0.499	0.57	0.495
I believe that the vaccination has efficacy to protect (Yes = 1, otherwise = 0)	0.89	0.314	0.87	0.338
I am well-communicated about the vaccination (Yes = 1, otherwise = 0)	0.76	0.427	0.79	0.410
I felt religious constraints to accept vaccination (Yes = 1, otherwise = 0)	0.49	0.500	0.62	0.485
I believe there remains a conspiracy against the vaccination (Yes = 1, otherwise = 0)	0.57	0.496	0.64	0.481
I have political pressure not to participate in vaccination (Yes = 1, otherwise = 0)	0.15	0.361	0.24	0.425
I have a trust on the vaccination process (Yes = 1, otherwise = 0)	0.90	0.303	0.87	0.338
I received many rumours about COVID-19 vaccination (Yes = 1, otherwise = 0)	0.66	0.474	0.62	0.487
I have adequate information on COVID-19 vaccination (Yes = 1, otherwise = 0)	0.79	0.411	0.79	0.406
GENDER	0.52	0.500	0.48	0.500

### Model summery

Table 3 summarises the models of both university groups. The joint impact of all predictor variables on the dependent variable was determined using the Nagelkerke R square test, which explains the model's result.

**Table 3.** Comparative model summery

Model summary					
−2 Log likelihood		Cox and Snell *R*^2^		Nagelkerke *R*^2^	
Public	Private	Public	Private	Public	Private
240.619^a^	296.087^a^	0.189	0.207	0.364	0.394

The results of the Cox and Snell R Square test indicate that the outcome variable was explained by the predictor variables used in the PuU and PrU samples to 18.9–36.4% *vs.* 20.7–39.4%, respectively, which are assumed to be good levels.

### Goodness of model fit

In Table 4, the significance level (*P* value) for the omnibus tests of the model coefficients is significant (*P* *<* *0.05*), while it was insignificant (*P* > 0.05) for the Hosmer and Lemeshow tests for both study models. These results indicate the very good model fitness of the study samples subjected to binary logistic regression.

**Table 4.** Omnibus tests of model coefficients and Hosmer and Lemeshow test

Omnibus tests of model coefficients						
*χ*^2^			df		Sig.	
Public		Private	Public	Private	Public	Private
Step	96.408	133.253	10	10	0.000	0.000
Block	96.408	133.253	10	10	0.000	0.000
Model	96.408	133.253	10	10	0.000	0.000
Hosmer and Lemeshow Test						
*χ*^2^			df		Sig.	
Public		Private	Public	Private	Public	Private
6.123		10.336	8	8	0.525	0.242

### Results of binary logistic regression analysis

Table 5 displays the comparative results of the regression analysis. According to the results, out of 10 predictors, ‘safety’ and ‘efficacy’ showed highly significant positive associations with vaccine acceptance in both cohorts (*P* *=* *0.000, P* *=* *0.005)*. ‘Political roles’ was found to have a significant (*P* *=* *0.02)* negative and a significant positive (*P* = 0.002) association with vaccine acceptance in public and private students, respectively. Additionally, ‘communication’ (*P* *=* *0.003)* and ‘trust’ *(P* *=* *0.01)* were found to have significant positive associations for PrU students, while ‘rumours’ (*P* *=* *0.03)* had negative association in PuU students Table 5: Binary logistic regression analysis for comparative model

**Table 5.** Binary logistic comparative models

Public university							
Variable	*B*	s.e.	Wald	Sig.	Exp(B)	95% CI for EXP(B)	
						Lower	Upper
Constant	−1.081	0.519	4.338	0.037	0.339		
Safety	1.671**	0.421	15.769	0.000	5.317	2.331	12.130
Side effect	0.537	0.400	1.799	0.180	1.711	0.781	3.750
Efficacy	1.966**	0.456	18.578	0.000	7.142	2.921	17.460
Communication	−0.248	0.434	0.327	0.567	0.780	0.333	1.826
Religiosity	0.393	0.406	0.936	0.333	1.481	0.668	3.285
Conspiracy beliefs	0.265	0.390	0.462	0.497	1.303	0.607	2.798
Political roles	−1.092*	0.484	5.086	0.024	0.336	0.130	0.867
Trust	0.112	0.508	0.048	0.826	1.118	0.413	3.028
Rumours	−0.766*	0.357	4.598	0.032	2.152	1.068	4.335
Information sufficiency	−0.298	0.421	0.501	0.479	0.742	0.325	1.694
Variable	*B*	s.e.	Wald	Sig.	Exp(B)	95% CI for EXP(B)	
						Lower	Upper
Private university							
Constant	−1.819	0.485	14.077	0.000	0.162		
Safety	1.297**	0.364	12.723	0.000	3.659	1.794	7.464
Side effect	0.328	0.333	0.968	0.325	1.388	0.722	2.667
Efficacy	1.108**	0.391	8.027	0.005	3.030	1.407	6.522
Communication	0.987***	0.331	8.886	0.003	2.684	1.402	5.136
Religiosity	0.613	0.322	3.615	0.057	1.845	0.981	3.470
Conspiracy beliefs	−0.218	0.321	0.463	0.496	0.804	0.429	1.507
Political roles	1.509***	0.498	9.194	0.002	4.523	1.705	11.997
Trust	1.041**	0.420	6.147	0.013	2.833	1.244	6.454
Rumours	0.140	0.331	0.180	0.671	1.151	0.602	2.200
Information sufficiency	−0.059	0.348	0.028	0.866	0.943	0.476	1.866

note: ** = significant at <0.01, * = significant at <0.05, level of significance.

### Pearson's *χ*^2^ test results

Table 6 shows the results of the Pearson's chi-squared test and odds ratio for risky group estimations. The odds of accepting the COVID-19 vaccine were 1.5 *vs.* 0.9 in PuU and PrU students, respectively, and both results were found to be insignificant (*P* *>* *0.05)* according to the *χ*^2^ test. Hence, statistically speaking, no group was identified as a vaccine-hesitant risk group among the university students in Bangladesh.

**Table 6.** Results of Pearson's *χ*^2^ test in the comparative model

Public university cohort				
*χ*^2^ tests				
	Value	Asymptotic significance (2-sided)	Exact sig. (2-sided)	Exact sig. (1-sided)
Pearson *χ*^2^	2.406^a^	0.121		
Continuity correction^b^	1.981	0.159		
Likelihood ratio	2.411	0.121		
Fisher's exact test			0.150	0.080
Linear-by-linear association	2.401	0.121		
*N* of valid cases	461			
Risk estimate		Value	95% CI	
			Lower	Upper
	Odds ratio for gender: (Female / Male)	1.566	0.886	2.770
	For cohort I intend to accept vaccination anytime = No	1.485	0.897	2.457
	For cohort I intend to accept vaccination anytime = Yes	0.948	0.886	1.015
	*N* of valid cases	461		
Private university cohort				
*χ*^2^ tests				
	Value	Asymptotic significance (2-sided)	Exact sig. (2-sided)	Exact sig. (1-sided)
Pearson *χ*^2^	0.071^a^	0.790		
Continuity correction^b^	0.019	0.889		
Likelihood ratio	0.071	0.790		
Fisher's exact test			0.801	0.444
Linear-by-linear association	0.071	0.790		
*N* of valid cases	573			
Risk estimate		Value	95% CI	
			Lower	Upper
	Odds ratio for gender: (Female / Male)	0.935	0.569	1.537
	For cohort I intend to accept vaccination anytime = No	0.943	0.610	1.457
	For cohort I intend to accept vaccination anytime = Yes	1.008	0.948	1.073
	*N* of valid cases	573		

## Discussion

The COVID-19 pandemic has destroyed not just the economy, health system and transport system, but the education system has also been very badly affected. Despite a widespread discussion about the effects of the pandemic on the economy and health systems, the catastrophic impact of the coronavirus on education systems in developing countries has yet to draw the attention of the world's community to a sufficient extent. Most developed nations have succeeded in overcoming the disastrous impacts of COVID-19 through the online development of traditional education systems and rapid vaccination coverage amongst students, because the ratio of vaccine coverage is higher in developed countries.

In this comparative study, we investigated the differences in intention to be vaccinated against COVID-19 between public and private university students in a developing country, and compared the potential factors influencing vaccine acceptance and hesitancy to help develop health policy. According to the study result, COVID-19 vaccine acceptance among the public and private university students was 88.1% and 87.6%, respectively. Survey studies conducted in Bangladesh have reported 72.7% and 72.3% COVID-19 vaccine uptake willingness among university students [26, 27]. Abedin *et al*. (2021) performed an empirical study, and reported a 74.6% COVID-19 vaccine acceptance rate among Bangladeshi young adults [19]. We collected data after the vaccination drive had begun throughout the country, and much of the populations were concerned about vaccination data. Moreover, the prevalence of vaccine hesitancy was high among older people, the less educated, day-labourers and chronically diseased individuals [24], while vaccine acceptance intention was high in students [17] and young adults [18] in Bangladesh. Globally speaking, an 89.4% positive intention to receive a COVID-19 vaccine was found among medical students in India [30], and 75% of university students agreed to take the COVID-19 vaccine in Kuwait [31].These results are consistent with our findings.

Low vaccine uptake intention and vaccine apprehension are complex heterogeneous events that were found to have increased by 90% since 2014 [32], and vaccine hesitancy was also found in the public in relation to COVID-19 vaccinations [10]. According to the regression model, ‘safety’ and ‘efficacy’ had highly significant and positive associations with vaccine uptake intention in both PuU and PrU students. It is evident that COVID-19 vaccine uptake intention is a dynamic phenomenon; vaccination willingness depends on the pandemic context, perceived community threats, health risks and concerns around the safety and efficacy of vaccines [33]. The delay in receiving a COVID-19 vaccine is related to confirmation about the safety and efficacy of COVID-19 vaccines in Bangladesh [15, 22, 24]. In the global context, 46% of college students showed concern about vaccine safety and efficacy when COVID-19 developed in Qatar [34]. Adequate information on vaccine safety, side effects and efficacy could ameliorate public confidence, and encourage them to get vaccinated against COVID-19 in Bangladesh [14, 16, 25].

According to our results, political roles had a significant influence on COVID-19 vaccine acceptance in both student cohorts. Political party membership has been shown to influence COVID-19 vaccine acceptance willingness, which could be considered a potential target for public health interventions [35].The political motives of students in underdeveloped countries are frequently regulated by the movements of national politics due to the socio-economical and cultural conditions [36].Students have a proud history of political engagement in Bangladesh [37], and students who are politically minded ought to be encouraged to voice their concerns and decisions regarding regional and national issues.

Alongside publicly funded universities, the privatisation of higher education in Bangladesh has opened up opportunities for the most financially capable students, along with meritorious individuals. The students from private universities referred to similar perceptions regarding the issue of political thoughts in Bangladesh; however, students of privately owned universities are reluctant to involve themselves with student politics that put party interests first. Several issues discourage PrU students from engaging in politics and move them away from political unions [38]. These students are more devoted to academic-and career-oriented behaviour. In contrast, most of the students, teachers, and offce employees of public universities are often engaged in political activities and the pursuit of partisan political goals. Students are encouraged to take part in political movements, and students become the frontline of politics inside the university. As a result, a political role was negatively associated with the vaccination decision in the PuU cohort. Political affiliations with opposition parties were recognised as a predominant factor of COVID-19 vaccine hesitancy in Bangladesh [20]. In the global context, individuals who received information on COVID-19 vaccines from political bodies became confused in making vaccination decisions [39]. In our study, ‘communication’ and ‘trust’ showed significant associations with COVID-19 vaccine acceptance among PrU students in Bangladesh. It has been reported that credible and culturally informed health communication influences health behaviour, guides decision making, helps address health concerns, and builds trust in the ability to deal with a pandemic (with the example of H1N1) [40]. The most critical predictor for converting vaccines to vaccinations and ensuring mass immunisation against COVID-19 in rural Bangladesh is health communication [25]. Health communication has been recognised as one of the key predictors of COVID-19 vaccine acceptance among Bangladeshi people [41, 42]. Poor trust and confidence in the country's health system lead to COVID-19 vaccine hesitancy among adults in Bangladesh [19]. Vaccine safety and efficacy are highly influenced by trust – in fact, trust was one of the key determinants of H1N1vaccine optimisation [40]. Consequently, trust has been identified as one of the potential drivers of COVID-19 vaccine acceptance among 89% of students in Romania [43], 73.6% of students in Nigeria [44], and 32.1% of students in multi-ethnic areas [45]. Furthermore, trust was an overarching issue in the global context of COVID-19 vaccine hesitancy among students hub[46].

Rumour has been identified as a contributor to COVID-19 vaccine decision-making among university students in Bangladesh. Since the country-wide vaccination process has started, a significant portion of people have been confused as to whether they should take the vaccine or not. Different rumours have been propagated about the origins of vaccines, and a smear campaign was established to embarrass the government. Many groups intentionally aired different forms of propaganda about the vaccine's origin. Several political parties discouraged people from taking the vaccine, and disseminated fake messages on vaccines. Kanozia and Arya [47] found that fake news and rumours were important issues in the context of COVID-19 vaccine decisions in India, Pakistan and Bangladesh. Previous research deduced that rumour was a barrier to mass COVID-19 vaccination in Bangladesh [25, 41]. Doubtful attitudes towards vaccines and anti-vaccination beliefs, such as those connected to conspiracies and religion, have been recognised as critical concerns in the global [18, 45] as well as national [12] context; however, anti-vaccination beliefs have been identified as insignificant predictors in this study. Since university students are well-equipped with access to scientific information, anti-vaccination beliefs could not amplify their vaccination sentiments. An advanced roadmap to conveying accurate, scientific and sustainable information via strategic communications could help build public trust in vaccination [48], and restoring public trust could nurture vaccine confidence by reducing anti-vaccination beliefs [49]. Despite the fact that few studies identified side effect as one of the potential determinants of COVID-19 vaccine acceptance [16, 21–23] in Bangladesh, in our study, side effects were insignificantly associated with vaccine acceptance. The aforementioned studies [16, 21–23] were conducted at the beginning of the vaccination programme when country-wide vaccination had not been started. Mild symptoms were observed within 48 h of the first dose; however, no severe adverse effects were found among the vaccinated [50]. Elderly vaccinated individuals and those with co-morbidities did not report any severe adverse effects after receiving the vaccine. Meanwhile, the government of Bangladesh enforced a country-wide mass vaccination programme on February 7,2021 based on Covishield, the Oxford–Astra Zeneca Covid-19 vaccine manufactured by the Serum Institute of India. To achieve basic nationwide vaccination coverage, the regulatory authority of the Directorate General of Drug Administration of Bangladesh approved seven vaccine candidates for use in the Bangladeshi population, and the Moderna COVID-19 vaccine was the last candidate included in the platform for emergency use. The government decided to administer COVID-19 vaccines free of cost to ensure mass vaccine coverage. The long-term persistence of COVID-19 has caused the education system to suffer collateral damage, adding to the woes of an already hard-hit sector and its students. Health policy makers have developed a strategy to include people from the higher educational sector in the vaccination programme on a priority basis, in order to enable them to resume regular classroom activities, and thereby encourage them to comply with government decisions [51]. In this comparative study, we collected data from a large sample to ensure the external validity and representativeness of the study's findings. In total, one thousand and thirty four students from public and private universities participated in this study. The variations in the respondents' demography and sample size strengthen our ability to generalise the study's results when addressing the mass population, and help in delivering a health messages that will increase public support for COVID-19 vaccination.

This study has practical implications for policy, practice and future research. The findings largely benefit policy makers, health stakeholders and vaccine promoters, in helping to develop evidence-based vaccine promotional strategies. Identifying potential factors underlying vaccine acceptance and hesitancy would be useful in developing rigorous public health interventions to combat the pandemic. The study findings will help us to overcome vaccination barriers, while facilitating nationwide vaccine rollout, and they will help the government to design immunisation protocols accordingly. In further research, this study could act as scientific evidence for initiating further observational studies of COVID-19 vaccine acceptance by examining the relationship between other confounding variables. Since the pattern of COVID-19 vaccine reluctance can alter over time [52], this study should be followed by long-term surveillance studies for tracking the temporal changes in factors associated with global COVID-19 acceptance.

This study has some limitations. The foremost limitation is that it used convenience sampling, so the results are prone recall and selection bias. The study thus did not involve the largest sample size, and so representation of university students was not adequate. Additionally, a non-response bias is a possibility, as those who did not respond might have been more vaccine-intentional or -hesitant in the context of COVID-19 than the study's respondents. This non-response could thus undermine the findings on the prevalence of COVID-19 vaccine hesitancy among students, resulting in larger differences between those who are willing to receive vaccine and those are not willing. This study identified and compared the potential factors influencing vaccine acceptance, which may differ between socio-psychological and behavioural contexts. With the frequent changes in the perceived health risks associated with the disease context, as well as the approval and deployment of COVID-19 vaccines themselves, individuals' behavioural perspectives and the pattern of COVID-19 vaccine reluctance could differ among young adults [52]. Therefore, it was difficult to predict the vaccine acceptance and hesitancy levels. Additionally, there are more factors associated with COVID-19 vaccine acceptance that remain unrecognised in this study.

## Conclusions

As a socially influential group, understanding university students' perspectives on COVID-19 vaccine acceptance, and expanding their awareness of vaccine readiness, are essential, because students are more vulnerable due to their active lifestyles and perceptions of invulnerability. This study reflects high COVID-19 vaccine acceptance in Bangladeshi university students. The comparative analysis shows that, several factors were associated with vaccination acceptance decisions, however, discrepancies among the potential factors were observed between public and private university students. The study concludes that safety, efficacy, political roles, communication, trust and rumour are the six factors most significantly associated with vaccine acceptance and hesitancy among university students in Bangladesh. Following a further breakdown, vaccine safety, efficacy and political roles were found to be significant for both groups of university students. Although communication and trust were identified as important positive determinants of COVID-19 vaccine acceptance in PrU students, rumour was found to have a negative effect on PuU students' vaccine acceptance. Public perceptions are likely to be changed as more vaccine-related safety and efficacy data become publicly available, thus conveying information to people through proper communication and trustworthy channels. Individualised publicity and education, combined with multidisciplinary interventions, are the preferred approach to improving students' adherence, attitudes and knowledge about COVID-19 vaccination's consequences. The patterns of COVID-19 vaccine pattern can alter over time; hence, long-term surveillance studies may be adopted to track the temporal changes in factors influencing COVID-19 vaccination. The current study's findings can support the government and health policymakers in implementing mass vaccination among university students in near-real-time.

## Data Availability

This manuscript does not contain any associated data; however the raw data that supports the finding of the manuscript are available upon reasonable request to corresponding author or first author.

## Supplementary material

For supplementary material accompanying this paper visit https://doi.org/10.1017/S0950268822001820. click here to view supplementary material
